# Use of intrapulmonary administration of thrombin in hematological malignancy patients with alveolar haemorrhage: A case series

**DOI:** 10.1097/MD.0000000000020284

**Published:** 2020-05-15

**Authors:** Jongmin Lee, Chin Kook Rhee, Seok Chan Kim, Young Kyoon Kim, Hee Je Kim, Seok Lee, Seok-Goo Cho, Jong Wook Lee

**Affiliations:** a Division of Pulmonary, Allergy and Critical Care Medicine, Department of Internal Medicine; b Division of Hematology, Catholic Blood and Marrow Transplantation Center, Seoul St. Mary's Hospital, College of Medicine, The Catholic University of Korea, Seoul, Republic of Korea.

**Keywords:** active bleeding, alveolar hemorrhage, hematological malignancy, intrapulmonary thrombin

## Abstract

**Introduction:**

Alveolar hemorrhage (AH) is characterized by the acute onset of alveolar bleeding and hypoxemia and can be fatal. Thrombin has been widely used to achieve coagulation and hemostasis. However, the efficacy of thrombin in patients with AH is unclear. Thus, this study aimed to evaluate the efficacy of thrombin administration in patients with hematological malignancy and AH.

**Patient concerns and diagnoses:**

This retrospective study included 15 hematological malignancy patients (8 men and 7 women; mean age 47.7 ± 17.3 years) with AH who were administered intrapulmonary thrombin between March 2013 and July 2018.

**Interventions and outcomes:**

All patients received bovine-origin thrombin (1000 IU/ml, Reyon Pharmaceutical Co., Ltd., Seoul, Korea) via a fiberoptic bronchoscope. A maximum of 15 ml of thrombin was injected via the working channel to control bleeding. The ability of thrombin to control bleeding was assessed. Additionally, the change in the PaO_2_/FiO_2_ (PF) ratio after intrapulmonary thrombin administration was evaluated. Intrapulmonary thrombin was administered a minimum of 3 days after starting mechanical ventilation in all patients, and it immediately controlled the active bleeding in 13 of 15 patients (86.7%). However, AH relapse was noted in 3 of the 13 patients (23.1%). The PF ratio improved in 10 of 15 patients (66.6%), and the mean PF ratio was significantly higher after thrombin administration than before administration (*P* = .03). No adverse thromboembolic complications or systemic adverse events were observed.

**Conclusion:**

Thrombin administration was effective in controlling bleeding in hematological malignancy patients with AH. Intrapulmonary thrombin administration might be a good therapeutic option for treating AH.

## 1 Introduction

Alveolar hemorrhage (AH) is a potentially fatal clinical syndrome that is characterized by acute onset of alveolar bleeding and hypoxemia. It is one of the most common complications among patients with hematologic malignancies, especially patients suffering from leukemia who received an allogeneic hematopoietic stem cell transplantation (HSCT). AH has a reported incidence of 3% to 20% in patients receiving HSCT and a mortality rate that ranges from 50% to 80%.^[1,2]^ The etiology of AH in patients with hematological malignancy is diverse and is typically associated with thrombocytopenia, infectious disease, allogeneic HSCT, and coagulopathies. Conventionally, the diagnosis of AH is based on a finding of abnormally increased blood content in fluid samples obtained during bronchoalveolar lavage (BAL) in the absence of signs of infection.^[3]^ In addition to supportive care, systemic corticosteroids have been used to treat AH in patients with hematological malignancies. So far, however, no prospective, randomized trials have investigated the role of systemic corticosteroids in patients with AH. Furthermore, recent studies have reported similarities in the clinical presentation, management, and high mortality rates in the presence and absence of infection (infection-associated AH [IAH] and diffuse AH [DAH], respectively).^[4,5]^ The difficulty of clinically distinguishing DAH from IAH raises concern that high-dose corticosteroids may increase the risk of infection.

Traditionally, to enhance pulmonary hemostasis and decrease the dose and duration of corticosteroid therapy, antifibrinolytic drugs such as tranexamic acid (TXA) and epsilon aminocaproic acid (EACA) have been suggested.^[6,7]^ However, a previous study reported that TXA did not improve mortality due to bleeding in patients with acute promyelocytic leukemia.^[8]^ EACA has also been used in addition to corticosteroids in patients with post-transplant AH; however, the effect of EACA in AH is controversial.^[7,9]^ Recently, the use of recombinant factor VIIa (rFVIIa) in patients with AH has increased. Although rFVIIa was originally developed for patients with hemophilia and acquired coagulation factor inhibitors, the off-label use of rFVIIa has increased and many studies have reported the impact of rFVIIa in the treatment of uncontrolled AH in patients with hematological malignancy.^[10,11,12,13]^ However, rFVIIa is prohibitively expensive,^[14,15]^ and the off-label use of rFVIIa has been associated with major thrombotic complications.^[15,16]^

Thrombin has been widely used to achieve coagulation and hemostasis. In the setting of hemoptysis, topical application of thrombin has also been employed.^[17,18]^ Thus far, however, few studies have investigated the effect of thrombin in patients with AH. We present our experience of the intrapulmonary administration of thrombin as a simple and effective technique for the treatment of AH in hematological malignancy patients in the intensive care unit (ICU).

## 2 Methods

### 2.1 Study population

In this retrospective study, we reviewed 15 hematological malignancy patients with AH who were treated with intrapulmonary administration of thrombin from March 2013 to July 2018. Patients were identified in 3 different ICUs at Seoul St. Mary's Hospital, Seoul, Republic of Korea. The diagnosis of AH was made based on the occurrence of all of the following clinical criteria:

1. acute onset of hypoxemia,
2. presence of diffuse pulmonary infiltrates on a chest X-ray or a computed tomography scan, and
3. confirmed bronchoscopically after consecutive BAL aliquots yielded an increasingly bloody return.^[19]^

The Acute Physiology and Chronic Health Evaluation II (APACHE II) and Sequential Organ Failure Assessment (SOFA) scores were calculated based on the vital signs, mental status, and laboratory findings of enrolled patients on the day of ICU admission. The Ethics Committee of Seoul St. Marys Hospital approved this study. The ethics review board waived the need to obtain informed consent due to the retrospective study design (KC18ZESI0804).

### 2.2 Bronchoscopic technique

Bronchoscopic procedures were performed at the bedside within the ICU by staff members with a minimum of 5 years clinical experience of bronchoscopy in the pulmonary division. Before the procedure, the patient was subcutaneously administered 0.5 mg of atropine sulfate to reduce oral secretions and bronchospasm. Before intrapulmonary thrombin instillation, BAL was performed to diagnose AH and conduct a microbiological investigation. The use of suction was meticulously avoided throughout the procedure. An endoscopic revision of the whole bronchial tree was made once the bleeding had been controlled. For intubated patients, the ventilator settings were generally set on a control mode with 100% FiO_2_ and PEEP ≥10 at the time of administration. Non-intubated patients received oxygen supplements by nasal probe or by mouth.

### 2.3 Therapy for AH

To enhance pulmonary hemostasis, patients received platelet transfusions in an attempt to achieve a level >50 × 10^9^/L, and fresh-frozen plasma to normalize prothrombin time and activated partial thromboplastin time. Intrapulmonary instillation of thrombin was performed in patients who did not improve despite receiving conventional treatment for >2 days. Conventional treatment was defined as high-dose corticosteroid (methylprednisolone 500 mg/day) and supportive care (e.g., ventilatory support, transfusion of blood products, infusion of tranexamic fluid, and antimicrobial therapy). All patients were treated with the instillation of bovine origin thrombin (1000 IU/ml, Reyon Pharmaceutical Co., Ltd., Korea) via a fiberoptic bronchoscope (FOB). The dosing and the frequency of thrombin instillation were at the discretion of the attending physician. Because there are no guidelines for intrapulmonary thrombin instillation, the dosing of thrombin was varied; to control bleeding, a maximum of 15 ml of 1000 IU/ml thrombin was injected at each lobe. The medication was administered by syringe via the working channel.

### 2.4 Statistical analysis

Statistically significant differences between groups were analyzed using the Wilcoxon signed rank test. Data are expressed as the mean ± standard deviations, median (interquartile range), and numbers and percentages. The distribution of continuous variables was determined using the Shapiro–Wilk normality test. A two-tailed *P* value <.05 was considered statistically significant. All statistical analyses were performed, and a graph was plotted using the R 3.4.1 version (R Foundation, Vienna, Austria).

## 3 Results

The characteristics of the 15 hematological malignancy patients with AH who received intrapulmonary thrombin instillation are provided in Table 1. The mean age of the patients was 47.7 ± 17.3 years, and 8 patients (53.3%) were men. The mean white blood cell count, absolute neutrophil count, and platelet count were 7.7 × 10^9^/L (1.3–18.3), 1.2 × 10^9^/L (0.3–3.0), and 43.9 ± 25.6 × 10^9^/L. The mean APACHE II and SOFA scores were 21.3 ± 7.0 and 8.0 ± 2.0. The underlying hematological diseases of the patients were acute myeloid leukemia (n = 7), acute lymphoblastic leukemia (n = 2), aplastic anemia (n = 1), non-Hodgkins lymphoma (n = 3), and myelodysplastic syndrome (n = 2). The reasons for ICU admission were respiratory failure (n = 10), septic shock (n = 2), massive hemoptysis (n = 2), and severe graft-versus-host disease (GVHD, n = 1). Among the 15 patients, 5 received allogeneic HSCT and 10 did not.

**Table 1:**
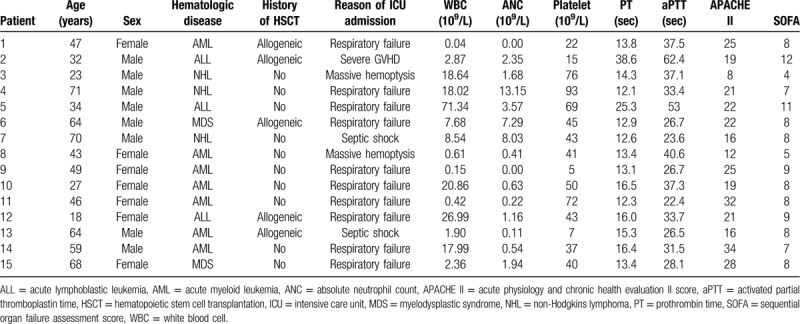
Baseline characteristics of patients with alveolar hemorrhage who received intrapulmonary thrombin instillation.

**Figure 1:**
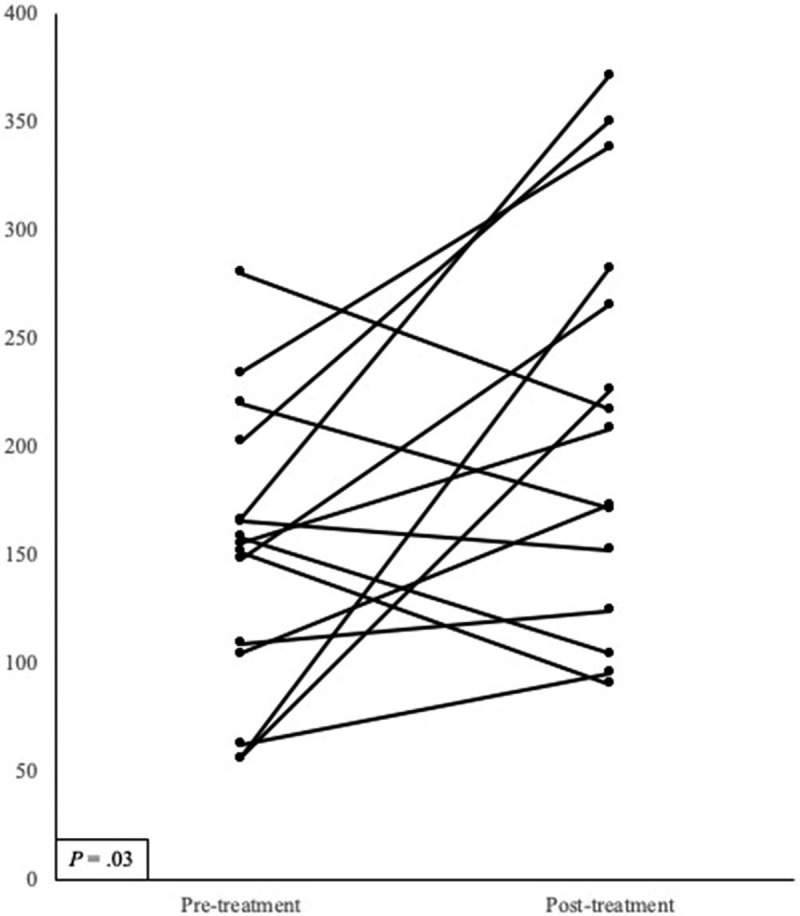
Change in the PaO_2_/FiO_2_ ratio after intrapulmonary instillation of thrombin. The mean of PaO_2_/FiO_2_ ratio at post-treatment was significantly higher than that at pre-treatment (151.1 ± 66.2 vs 211.1 ± 93.7, *P* = .03).

Table 2 presents the results of intrapulmonary thrombin instillation. All patients received high-dose systemic steroids before intrapulmonary thrombin instillation; 14 of 15 patients also received tranexamic fluid infusion. Intrapulmonary thrombin instillation was performed a minimum of 3 days after starting mechanical ventilation in all patients. Intrapulmonary instillation of thrombin immediately controlled the active bleeding in 13 of 15 (86.7%) patients. However, AH relapse was observed in 3 of 13 (23.1%) patients. In the cases of patients 2 and 4, the AH continued despite the intrapulmonary thrombin instillation. In patient 10, AH relapse occurred 5 days after the thrombin instillation and necessitated bronchial artery embolization. After bronchial artery embolization, the AH was controlled and the patient survived. Bronchoscopic examination could be completed in all patients, and there were no thrombotic complications or complications that required its suspension after thrombin instillation. Figure 1 illustrates the change in the PaO2/FiO2 ratio (PF ratio) after intrapulmonary thrombin instillation. The post-procedure PF ratio was measured at day 2 post-procedure. After intrapulmonary thrombin instillation, the PF ratio was improved in 10 of 15 patients and the mean PF ratio was significantly improved (151.1 ± 66.2 vs 211.1 ± 93.7, *P* = .03).

**Table 2:**
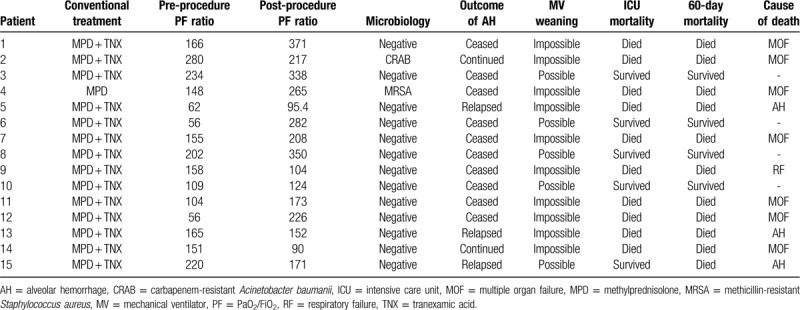
Outcome of patients with AH who received intrapulmonary instillation of thrombin.

The etiologies of AH differed among the patients, and microorganisms were isolated from BAL fluid in 2 patients: carbapenem-resistant *Acinetobacter baumanii* (patient 2) and methicillin-resistant *Staphylococcus aureus* (patient 4). Co-infections with cytomegalovirus, one of the main infectious diseases that cause AH, and other respiratory viruses were not found in any patient.

Among the 15 patients, 11 eventually died. Multiple organ failure was the leading cause of death (n = 7), and 3 patients died of respiratory failure due to AH (Table 2). In the case of patient 15, AH was improved after the intrapulmonary thrombin instillation and the patient was successfully weaned from the mechanical ventilator. However, at day 7 post-procedure, she experienced an AH relapse and died. Among a total of 4 survivors, 3 patients became long-term survivors with normal pulmonary function (patients 3, 6, and 10).

## 4 Discussion

While AH is a rare cause of death in patients with hematological malignancies, it is still a fatal clinical syndrome with a mortality rate of approximately 70%.^[20,21]^ In hematological malignancy patients with AH, acute lung injury occurs due to intra-AH and diffuse alveolar damage due to immunologic mechanisms.^[20,22]^ Therefore, anti-inflammatory and hemostatic therapies should be the cornerstones of therapy.

To improve pulmonary hemostasis, antifibrinolytic drugs such as TXA and EACA have been used alongside corticosteroids.^[6,7]^ TXA is a synthetic derivative of the amino acid lysine that binds to plasminogen and blocks its binding to fibrin and subsequent activation to plasmin.^[23]^ TXA in either an aerosolized form or as intrapulmonary injections has been reported to control bleeding in a few cases of pulmonary hemorrhage.^[6,24]^ EACA is another potent antifibrinolytic agent used to control bleeding by inhibiting the plasmin-plasminogen system.^[25,26]^ In the case of AH in patients with hematological malignancy, a retrospective study reported that EACA improved the mortality rate by 100 days compared to that in those treated with steroids alone.^[7]^ However, the effect of TXA and EACA in AH remains controversial.

In hematological malignancy patients with AH, high-dose corticosteroids have been widely used because the pathogenesis of AH is considered to be an inflammatory response to various insults.^[27]^ However, no prospective, randomized controlled trials have confirmed the efficacy of corticosteroids in patients with AH. Previous studies reported that the use of high-dose systemic corticosteroids resulted in transient clinical improvement.^[28,29]^ However, the use of high-dose corticosteroids has not improved the outcome of AH; previous studies have reported mortality rates of 70% to 100% in AH patients treated with high-dose corticosteroids.^[5,20,28,29,30,31]^

To date, there is no definite treatment for AH in patients with hematological malignancies. Therefore, there remains an urgent need for novel methods to treat AH. Recently, the use of rFVIIa has increased in patients with AH. rFVIIa is an analog of the naturally occurring protease and was originally developed for the treatment of hemophilia patients with acquired coagulation factor inhibitors.^[32]^ Several case reports have described the use of rFVIIa for the management of AH after HSCT.^[12,33,34]^ However, rFVIIa is prohibitively expensive, and off-label use of rFVIIa has been associated with major thrombotic complications.^[14,15,16]^

Thrombin is a key enzyme in hemostasis and thrombosis, and regulates pro- and anti-coagulant reactions by interacting with other coagulation proteins and cellular receptors.^[35]^ Thrombin production is the final coagulation step required to cleave fibrinogen into fibrin, which provides a hemostatic lattice for platelet aggregation and thrombus formation at the site of injury.^[36]^ In addition to the coagulation cascade, thrombin causes vasoconstriction at the smooth muscle cell level and promotes platelet aggregation at the site of the thrombus.^[36]^ Prior studies have reported the effect of thrombin or fibrinogen-thrombin in the treatment of severe hemoptysis with acceptable success rates.^[17,18,37,38]^ Thus far, however, no studies have investigated the effect of intrapulmonary thrombin instillation in patients with AH. To our knowledge, this is the first study to apply thrombin in the treatment of hematological malignancy patients with AH.

In this study, we have illustrated efficacy of intrapulmonary thrombin instillation in mechanically-ventilated patients with hematological malignancies who developed AH. Our findings indicate that intrapulmonary thrombin instillation can be a useful adjuvant therapy along with supportive care such as corticosteroids or TXA. Although ten patients (66.7%) died in the ICU and 11 patients (73.3%) died within 60 days of the onset of AH, thrombin instillation improved the respiratory status. Bleeding was controlled rapidly in 13 of 15 patients (86.7%) and oxygenation was significantly improved after the procedure. Furthermore, no adverse thromboembolic complications or systemic adverse events were observed. Although the majority of the initial presentations of AH in this study involved respiratory failure (66.7%), the most common cause of death was multiple organ failure (63.6%). This result is consistent with previous studies; respiratory failure with active pulmonary hemorrhage was responsible for <15% of the deaths.^[29]^

In summary, this is the first study to illustrate the efficacy of intrapulmonary thrombin instillation in patients with hematological malignancies who developed AH. Bleeding was controlled effectively and no systemic or thromboembolic adverse events were observed after intrapulmonary thrombin instillation. Intrapulmonary thrombin might be a good therapeutic option for the treatment of AH, particularly when standard treatment fails or bleeding is immediately life-threatening. Several important shortcomings and limitations of our study should be taken into account. First, as this was a retrospective study, it was impossible to compare the effect of intrapulmonary thrombin instillation in AH patients with a control group. Second, the sample size of the present study was small. Further studies with prospective design are needed, with a larger number of patients, to fully assess the risks, benefits, and optimal dosing strategy of thrombin as an adjunctive therapy in the treatment of AH. Despite these limitations, the main advantage of the present study is that it is the first to apply intrapulmonary thrombin in hematological malignancy patients with AH.

## Acknowledgments

The authors thank Minyoung Park at Seoul National University, for her helpful advice on the structuring of this manuscript.

## Author contributions

**Conceptualization:** Chin Kook Rhee.

**Data curation:** Jongmin Lee.

**Formal analysis:** Seok Lee.

**Methodology:** Seok Chan Kim.

**Resources:** Hee Je Kim.

**Supervision:** Seok-Goo Cho.

**Validation:** Young Kyoon Kim.

**Writing – original draft:** Jongmin Lee.

**Writing – review & editing:** Jong Wook Lee.
